# Caregiver report of the experiences of infant and child diaper insecurity, social support, and utilization of resources to help meet basic family need in the United States: A nationally-representative cross-sectional survey

**DOI:** 10.1177/17455057261456861

**Published:** 2026-05-30

**Authors:** Kelley E. C. Massengale, Megan V. Smith, Kristin P. Tully

**Affiliations:** 1 681930 National Diaper Bank Network, New Haven, CT, USA; 2 Department of Obstetrics and Gynecology, University of North Carolina at Chapel Hill, Chapel Hill, NC, USA

**Keywords:** diaper insecurity, diaper bank, diaper need, material hardship, material basic needs, maternal mental health

## Abstract

**Background:**

Diaper insecurity is associated with caregiver health and quality of life through relationships with financial stress, inability to meet other basic health and material needs, constraints on engagement in community, workforce, school, and early childhood education, and negative associations with caregivers’ own and their child’s health and mental health.

**Objective:**

The purpose of this study is to better understand affective components of diaper affordability, how these components may contribute to perceptions of social support, and to understand if and how households would like to receive diaper support.

**Design:**

Cross-sectional observational study.

**Methods:**

Data were collected via an online survey from March-April 2024. Surveys were completed by U.S. adults (N=1,000) who were caregivers of a child in their home wearing diapers.

**Results:**

Half of households (46.7%, n=467) reported diaper insecurity. Caregivers with diaper insecurity were more likely to report feeling alone (OR=8.19, CI=5.78-11.59), judged (OR=6.83, CI=5.07-9.21), helpless (OR=4.62, CI=3.54-6.03), and stressed or anxious (OR=4.54, CI=3.46-5.95) due to diaper affordability compared to those who reported being diaper secure. Most households with diaper insecurity (84.6%, n=395) had accessed resources for help meeting basic needs. Participants with diaper insecurity were less likely to feel treated in the way they deserve by healthcare professionals, community organizations, or the people close to them compared to participants without diaper insecurity. Diaper insecurity and negative affective components of diaper affordability significantly predicted perceived social support F (26, 972)=20.429, *p*< 0.01), adj. *R*^2^=.34. Overall, the majority of all caregivers (68.1%, n=639) reported that receiving help with diapers from a local diaper bank would be “extremely helpful” or “very helpful.”.

**Conclusion:**

More families in the United States may be struggling to afford diapers than are identified with diaper insecurity screening questions. Caregivers identified negative affective components of diaper affordability including feelings of judgement and aloneness which were associated with lower perceptions of social support. Households with and without diaper insecurity indicated that receiving 50 free diapers per month per child from a local diaper bank would be helpful. The support and expansion of diaper banks’ distribution efforts would help families with young children in ways they identified as meaningful.

## Plain language summary

**Why we did this study:**

Many families in the United States cannot afford enough diapers for their infants and young children which can be stressful for the adults and can be bad for children’s health.

**What we wanted to learn:**

We wanted to know if families who struggle to afford diapers have similar feelings to each other, what resources they turn to for help, if those resources support them, and if they would like to also receive diapers from those resources.

**What we did:**

In the spring of 2024, 1,000 families in the United States with babies and young children wearing diapers took a survey online. The survey asked questions about whether families could afford diapers, what resources helped them, and if and how they might want help with diapers.

**What we learned:**

Half of the families who took the survey had a hard time affording diapers. Families who could not afford enough diapers felt lonely, stressed, and judged. Most families said that they would like to have help with diapers from a diaper bank. Even families who could afford diapers said that it would be helpful to have help from a diaper bank.

**Why is this important:**

More families have a hard time affording diapers than people realize. Families who struggle to buy diapers feel alone because of it. Many people think of diaper banks as places in the community that can help them. Diaper banks need to have more money so that they can help more families.

## Introduction

Parenting young children can be simultaneously joyful and stressful.^1^ Caregivers with young children not only have many sources of stress, but are more likely to report high levels of stress than adults who are not parents.^2^ One specific stressor for caregivers of infants and young children that the United States health programs fail to universally address is the cost of diapers which may be as much as $125 (U.S. dollars) monthly per child.^3,4^ Families experience diaper insecurity when they cannot afford a diaper supply that allows for regular diaper changes at healthy intervals.^5^ Across the United States, 1 in 2 households experience diaper insecurity.^6–8^ Newly recognized as a social driver of health, diaper insecurity is associated with health outcomes and quality of life through multiple pathways and for multiple generations.^9,10^ These linkages include financial stress, inability to meet other basic health and material needs, constraints on engagement in community, workforce, school, and early childhood education, negative impacts on children’s physical and developmental health, and negative impacts on caregivers’ mental health.^3–14^

Support to address diaper insecurity is an important component of facilitating new families’ participation in workplace, caregivers’ continuing their education, early childhood opportunities, and caregiver mental health.^12^ Childcare providers typically require that families supply all of the diapers needed by children during the childcare day, even if childcare tuition is free or subsidized.^3^ If families cannot furnish the requisite diapers, adults may have to miss work or their own schooling or job training to care for their child at home instead.^3^ This has potential repercussions for take-home pay, workforce participation, disruptions to children’s routines and early childhood education, and caregivers’ mental health and well-being.^15^

In the United States, federally and state funded safety net programs exist to support some individuals and households in meeting basic needs which they may struggle to afford.^16^ These programs include Medicaid health coverage, Supplemental Nutrition Assistance Program [SNAP] and Supplemental Nutrition Program for Women, Infants and Children [WIC] food support, and Temporary Assistance for Needy Families [TANF] cash assistance among others.^16^ The absence of diapers in federal safety net programs has given rise to a nationwide infrastructure of nonprofit diaper banks via the National Diaper Bank Network.^17^ Diaper banks’ diaper distribution is linked to positive impacts on caregiver stress, child health, and the allocation of households’ limited financial resources to food purchases, living and healthcare expenses, and other material basic needs.^12,15^ When diaper banks distribute diapers via partnerships with other agencies offering resources and supports for families with infants and young children, diaper distribution is associated with families’ increased communication with agency staff, increased engagement in agency programming, and increased connections to address other unmet needs.^18^ Although diaper banks’ diaper distribution offers multiple benefits to families, not all communities have a diaper bank.^17,19^ To better understand families’ experiences with diaper affordability and to identify whether increased access diaper support would be helpful, the present study sought to address the following research questions.

1) What are the affective components of diaper affordability for caregivers of infants and young children? We hypothesized that caregivers experiencing diaper insecurity would be more likely to identify with negative affective statements related to diaper affordability than caregivers who were not experiencing diaper insecurity.
2) Are affective components of diaper affordability associated with perceptions of social support among caregivers of infants and young children? We hypothesized that negative affective components of diaper affordability would be associated with lower perceptions of social support.
3) Would households with young children like to receive support with diapers? We hypothesized that households experiencing diaper insecurity would be more likely to indicate that they would like to receive diaper support than households not experiencing diaper insecurity.

## Methods

### Data collection

Cross-sectional survey data were collected via an online survey fielded by YouGov from March to April 2024. YouGov, an international technology company, specializes in online research data and analytics.^20^ The survey questions were developed by the study authors and their colleagues. Then, the online survey was hosted by YouGov who recruited eligible participants from their web-based panel of adults to complete the survey. YouGov employes multiple monitoring strategies and quality control measures to promote survey data validity.^20^

U.S. adults were eligible to complete the survey if they met all of the following criteria: 1) lived with a child under age 4 years who wore any diaper or pull-up at any point throughout the day or night, 2) child under age 4 years was not yet toilet trained, 3) adult was responsible for changing the diapers of the children under age 4 years at least 25% of the time the child was at home, and 4) adult purchased the diapers, diaper wipes, diaper rash cream, etc. or their household at least some of the time. Caregivers were excluded from participation if they completed less than 25% of the diaper changes at home and/or could not read and complete the survey in English.

YouGov created a modeled frame of the U.S. adult population based on multiple sources including the 2020 Cooperative Election Study (CES) surveys, the 2020 Current Population Survey (CPS) Voting and Registration supplements, the 2020 National Election Pool (NEP) exit poll, public records of voter files, and the American Community Survey (ACS) public use microdata.^20^ A sampling frame was then built to match the modeled frame such that the survey respondents were nationally representative of the U.S. adult population with regard to the demographic variables of gender, race, age, income relative to family size, and educational attainment.^20^ The survey weighting methodology is available online from YouGov.^20^ Thus, N=1,000 adult caregivers with shared or primary diaper changing and purchasing responsibilities for a child in their home younger than age four composed the final sample, nationally representative of adults in the U.S. The online survey included questions about caregiver characteristics, utilization of resources, and experiences with healthcare professionals. Demographic data measured included gender, United States region, highest education level attained, racial and ethnic identity, and household income. The authors developed utilization and experience survey items from peer-reviewed literature and subject matter expertise.^6,7,21–23^ A group of n=22 respondents participated in a pilot of the survey questions. Participants could skip any survey question they did not wish to answer.

### Measures

#### Affective components of diaper affordability

Caregivers were asked for their agreement or disagreement with a series of statements about diaper affordability to determine whether they have struggled, felt alone, judged, helpless, stressed, or anxious related to their experiences with diaper affordability. Responses of “completely agree” or “agree” were combined to create a binary variable for agreement or disagreement with each affective statement.

#### Diaper insecurity

Caregivers were considered to have experienced diaper insecurity if they reported that they “completely agree” or “agree” with one or more of the following statements about experiences diapering the children under age 4 years in their present household situation:

I do not always have enough diapers to change my child's diapers as often as I would like.

I find it difficult to afford buying diapers for the child(ren) in my household.

I have run out of diapers for the child(ren) in my household because I could not afford enough diapers.

#### Social support

Perceptions of social support were measured and scored utilizing the 12-item Interpersonal Support Evaluation List [ISEL-12].^21^ The scale measures components of appraisal, belonging, and tangible support.^21^ Scale scores range from 0 to 36 with 36 indicating high perceived support.^21^ The scale’s Cronbach’s alpha, α=0.82, demonstrates reliability among the scale components.^24^

#### Supportive interactions

Caregivers were asked the frequency (i.e., always, often, sometimes, rarely, or never) to which they were treated in the way that they deserved by healthcare professionals, community organizations, and the people close to them. They were also asked the frequency in which these same groups were positive or encouraging to them. A binary variable was created to assess differences between participants who felt reliably supported or encouraged (always or often) compared to those who did not (sometimes, rarely, or never).

#### Diaper support

Caregivers were asked for their interest or comfort in diaper support from a variety of sources including: guidance from a nurse or other health professional; assistance from family, friends, diaper banks, or charity; free diapers from a local diaper bank; or the expansion of federal and/or state programs to offer financial assistance for diaper purchases.

### Statistical analysis

IBM SPSS version 31.0 was used for statistical calculations.^25^ Categorial variables were assessed with descriptive statistics. Group differences between households experiencing diaper insecurity and those who did not experience diaper insecurity were evaluated using Chi-square tests, odds ratios, and an independent samples t-test. Multiple regression analysis assessed the relationship between perceptions of social support, diaper insecurity, and affective components of diaper affordability while controlling for having moved homes in the past year and the sociodemographic characteristics of racial and ethnic identity, household income, marital status, education completed, and employment status.

The majority (77.8%) of respondents were not missing any data to calculate their ISEL-12 scale score, the dependent variable in the multiple regression analysis. For the remaining participants, 13.6% were missing 1 variable, 2.9% were missing 2 variables, 5.7% were missing 3 or more variables. We employed mean substitution to impute missing variables for ISEL-12 scores.

We followed the Strengthening the Reporting of Observational Studies in Epidemiology (STROBE) checklist during manuscript preparation.^26^

## Results

### Caregiver characteristics

The majority of participants identified as female (56.8%, n=568), with white racial identity (54.9%, n=548), and had completed some formal education after high-school (61.0%, n=619) (Table 1). Participants with diaper insecurity were more likely than participants without diaper insecurity to identify with Hispanic ethnicity or to report the lowest range of household income. Additionally, participants with diaper insecurity were more likely to report household income in low-income (<200% federal-poverty level) or middle-income (200% federal-poverty level to $125,000) brackets.

**Table 1.** Caregiver characteristics.

Characteristic	Caregivers with diaper insecurity (n=467)	Caregivers without diaper insecurity (n=533)	All caregivers (N=1,000)
**Gender Identity**	​	​	***NS***
Female	53.0% (n=248)	60.2% (n=320)	56.8% (n=568)
Male	46.6% (n=218)	39.3% (n=209)	42.7% (n=427)
Non-binary	0.4% (n=2)	0.2% (n=1)	0.3% (n=3)
Another identity	0% (n=0)	0.4% (n=2)	0.2% (n=2)
**United States Region**	​	​	***NS***
Northeast	15.2% (n=71)	14.1% (n=75)	14.6% (n=146)
Midwest	20.8% (n=97)	25.0% (n=133)	23.0% (n=230)
South	37.0% (n=173)	37.0% (n=197)	37.0% (n=370)
West	27.0% (n=126)	23.9% (n=127)	25.3% (n=253)
**Education**	​	​	*X*^2^(5, N=1,000) =73.061, *p*<.001
No high school diploma	6.6% (n=31)	2.8% (n=15)	4.6% (n=46)
High school diploma	42.6% (n=199)	25.5% (n=136)	33.5% (n=335)
Some college	19.9% (n=93)	18.2% (n=97)	19.0% (n=190)
2-year college degree	10.3% (n=48)	10.1% (n=54)	10.2% (n=102)
4-year college degree	13.5% (n=63)	23.1% (n=123)	18.6% (n=186)
Post-undergraduate degree	7.1% (n=33)	20.3% (n=108)	14.1% (n=141)
**Racial and ethnic identity**	​	​	*X*^2^(7, N=1,000) =29.005, *p*<.001
American Indian/Alaska Native	1.1% (n=5)	0.4% (n=2)	0.7% (n=7)
Asian	3.2% (n=15)	7.5% (n=40)	5.5% (n=55)
Black	12.0% (n=56)	13.7% (n=73)	12.9% (n=129)
Hispanic	24.8% (n=116)	15.4% (n=82)	19.8% (n=198)
Middle Eastern	.4% (n=2)	.4% (n=2)	.8% (n=4)
Multiple identities	6.0% (n=28)	4.1% (n=22)	5.0% (n=50)
White	51.4% (n=240)	57.9% (n=308)	54.9% (n=548)
Another identity	1.1% (n=5)	0.6% (n=3)	0.8% (n=8)
**Income Group**	​	​	*X*^2^(2, N=1,000) =93.473, *p*<.001
Low-income (<200% federal-poverty level)	59.7% (n=279)	30.8% (n=164)	44.3% (n=443)
Middle-income (200% federal-poverty level to $125,000)	30.4% (n=142)	43.2% (n=230)	37.2% (n=372)
High-income >$150,000	9.9% (n=46)	26.1% (n=139)	18.5% (n=185)

### Diaper insecurity experiences

Nearly half of households in the sample reported diaper insecurity (46.7%, n = 467) per the three screening questions. Most study participants (70.8%, n=708) endorsed that, “More families cannot afford diapers in the U.S. than people realize” (Table 2). Also, the majority (60.6%, n=606) indicated that, “Parents who struggle to afford diapers for their child (ren) are hesitant to discuss it.” Table 2 lists responses to items describing affective components of diaper affordability overall and by caregivers with and without diaper insecurity. There were differences between the groups with all questions asked, with greater odds of those with diaper insecurity indicating other parents/caretakers do not understand the struggles, feeling alone, negative judgement, feeling helpless, and feeling stressed or anxious related to affording diapers for their children.

**Table 2.** Caregiver agreement with statements about diaper affordability.

Agreement with statements about diaper affordability	Caregivers with diaper insecurity (n=467)	Caregivers without diaper insecurity (n=533)	All caregivers (N=1,000)	Odds ratio	95% confidence interval	*X*^2^ (df=1)	p-value
Other parents/caretakers don’t understand the struggles I have with affording diapers for my child (ren).	54.4% (n=254)	16.9% (n=90)	34.4% (n=344)	5.87	4.39 – 7.85	155.15	<.001
I feel alone because I cannot afford enough diapers for my child (ren).	44.8% (n=209)	9.0% (n=48)	25.8% (n=528)	8.19	5.78 – 11.59	166.58	<.001
I feel that other parents/caretakers are judging me as a bad parent/caretaker because I struggle to afford enough diapers for my child (ren).	55.0% (n=257)	15.2% (n=81)	33.8% (n=338)	6.83	5.07 – 9.21	176.52	<.001
More families cannot afford diapers in the U.S. than people realize.	77.9% (n=364)	64.5% (n=344)	70.8% (n=708)	1.94	1.47 – 2.57	21.63	<.001
I feel helpless when I cannot afford enough diapers for my child (ren).	68.3% (n=319)	31.8% (n=170)	48.9% (n=489)	4.62	3.54 - 6.03	132.6	<.001
Parents who struggle to afford diapers for their child (ren) are hesitant to discuss it with others or ask for help.	67.7% (n=316)	54.4% (n=290)	60.6% (n=606)	1.75	1.36 – 2.27	18.32	<.001
I feel stressed or anxious when I am unable to afford enough diapers for the child (ren) in my household.	74.1% (n=346)	38.6% (n=206)	55.2% (n=552)	4.54	3.46 – 5.95	126.43	<.001

## Resources accessed for family support

Caregivers reported whether they had utilized each of 12 different types of resources to help meet basic family needs anytime in the past 12 months for their household (Table 3). Overall, (63.4%, n=634) accessed one or more type of resource listed. Any reported use was higher among caregivers with diaper insecurity (84.6%, n=395) than those without diaper insecurity (44.8%, n=239). The odds of people accessing each resource was greater among those with diaper insecurity, with the most commonly accessed being SNAP, family members, WIC, and food banks. The least common sources of support utilized to help families meet their basic needs from the listed resources were hospitals or health clinics (4.3%, n=43) and homeless shelters (3.1%, n=31). Households with diaper insecurity accessed, on average, 2.65 (*SD*=2.05) different resources for help meeting basic needs in the past 12 months. Households without diaper insecurity accessed, on average, 1.20 (*SD*=1.65) different resources. The difference between the two groups was statistically significant, *t* (895) = 12.23, *p*<.001. Additionally, caregivers with diaper insecurity (80.1%, n=374) were more likely than caregivers without diaper insecurity (48.6%, n=259) to indicate that they “completely agree” or “agree” they would feel comfortable accepting public assistance (e.g. WIC, SNAP, or TANF) (*X*^2^ = 106.27, *p* <.001).

**Table 3.** Experiences of diaper insecurity and access of family supports to meet basic needs in the past year.

Supports accessed within past 12 months to meet basic needs for your family	Caregivers with diaper insecurity (n=467)	Caregivers without diaper insecurity (n=533)	All caregivers (N=1,000)	Odds ratio	95% confidence interval	*X*^2^ (df=1)	p-value
Accessed 1 or more family supports listed below	84.6% (n=395)	44.8% (n=239)	63.4% (n=634)	6.75	4.98 – 9.14	169.42	<.001
Women, Infants and Children (WIC)	34.9% (n=163)	17.1% (n=91)	25.4% (n=254)	2.60	1.94 – 3.50	41.76	<.001
Temporary Assistance for Needy Families (TANF)	9.9% (n=46)	4.9% (n=26)	7.3% (n=73)	2.13	1.30 – 3.51	9.21	.002
Supplemental Nutrition Assistance Program (SNAP)	44.3% (n=207)	21.2% (n=113)	32.0% (n=320)	2.97	2.25 – 3.91	61.47	<.001
Social Security Insurance (SSI)	13.7% (n=64)	4.3% (n=23)	8.6% (n=86)	3.52	2.15 – 5.77	27.63	<.001
Family member	36.8% (n=172)	22.5% (n=120)	29.2% (n=292)	2.01	1.52 – 2.65	24.68	<.001
Co-workers	9.4% (n=44)	2.3% (n=12)	5.6% (n=56)	4.52	2.36 – 8.66	24.21	<.001
Faith-based organizations/charities	16.9% (n=79)	5.3% (n=28)	10.7% (n=107)	3.67	2.34 – 5.76	35.44	<.001
Homeless shelters	6.2% (n=29)	0.4% (n=2)	3.1% (n=31)	17.58	4.17 – 74.08	28.21	<.001
Local community-based charities or nonprofit organizations	14.6% (n=68)	5.3% (n=28)	9.5% (n=95)	3.07	1.94 – 4.87	24.85	<.001
Diaper banks	15.6% (n=73)	5.8% (n=31)	10.4% (n=104)	3.00	1.93 – 4.66	25.74	<.001
Food banks	33.0% (n=154)	10.1% (n=54)	20.8% (n=208)	4.36	3.10 – 6.14	78.86	<.001
Hospitals or health clinics	6.6% (n=31)	2.3% (n=12)	4.3% (n=43)	3.09	1.57 – 6.08	11.64	<.001

### Social support

The overall average score of perceived social support, on a scale of 0-36 as measured by the ISEL-12 questionnaire, was *M*=22.0, (*SD*=7.4). The average total score among households with diaper insecurity (*M*=19.1, *SD*=6.9) was significantly lower compared to the average score among households without diaper insecurity (*M*=24.5, *SD*=6.8), indicating lower perceptions of social support (*t* (998) = -12.36, *p*<.001) among those families with diaper insecurity.

Additional components of social support were assessed by asking study participants how often they were treated in the ways deserved and/or treated in a positive or encouraging manner. Caregivers experiencing diaper insecurity were less likely to feel that healthcare professionals, community organizations, or the people close to them treated them in the ways they deserved or were positive or encouraging to them when compared to caregivers without diaper insecurity (Table 4).

**Table 4.** Caregiver feelings about interactions with healthcare professionals, community organizations, and people close to them.

Experience (How often…) (response of always or often)	Caregivers with Diaper Insecurity (n=467)	Caregivers without Diaper Insecurity (n=533)	All Caregivers (N=1,000)	Odds Ratio	95% Confidence Interval	*X*^2^ (df=1)	p-value
Do you feel treated by healthcare professionals in the way that you deserve?	71.4% (n=327)	85.4% (n=440)	78.8% (n=767)	.43	.31 - .59	28.63	<.001
Do you feel treated by community organizations in the way that you deserve?	68.2% (n=300)	84.7% (n=382)	76.5% (n=682)	.39	.28 - .54	33.85	<.001
Do you feel treated by the people close to you in the way that you deserve?	67.4% (n=312)	85.2% (n=445)	76.9% (n=757)	.36	.26 - .49	44.01	<.001
Are healthcare professionals positive or encouraging to you?	66.0% (n=299)	78.9% (n=408)	72.9% (n=707)	.52	.39 - .69	20.37	<.001
Are community organizations positive or encouraging to you?	60.1% (n=267)	77.2% (n=353)	68.8% (n=620)	.44	.33 - .59	30.71	<.001
Are the people close to you positive or encouraging to you?	65.7% (n=302)	82.2% (n=425)	74.4% (n=727)	.41	.31 - .56	35.03	<.001

Multiple regression analysis predicted perceived social support (ISEL-12) scores among caregivers while controlling for sociodemographic characteristics (Table 5). ISEL-12 scores measuring perceived social support significantly predicted diaper insecurity, regular contact with other parents of young children, multiple affective components of diaper affordability, and being treated respectfully or encouraged by people close to them *F* (26, 972) = 20.43, *p* < 0.01, adjusted *R*^2^ =.34. Statistically significant predictors of lower perceived social support scores included: diaper insecurity, feeling that other caregivers do not understand their struggles with diaper affordability, feeling judged as a bad caregiver for struggling to afford diapers, feeling alone due to diaper affordability, feeling helpless due to diaper affordability, feeling people close to them do not treat them respectfully, feeling people close to them are not positive or encouraging to them, having moved homes in the past year, and homemaker employment status. Statistically significant predictors of higher perceived social support scores included: regular contact with other parents of young children, feeling that people around them were willing to help their neighbors, Hispanic identity, and having completed a 4-year college degree as the highest educational attainment. **Comfort receiving diaper support**.

**Table 5.** Summary of multiple regression analysis for variables predicting perceived social support (ISEL-12) score among all caregivers (n =973), controlling for demographic variables.

Predictor	B	SE B	β	95% CI		
Diaper insecurity	-2.11 ***	.5	-.14	-3.06	-	-1.16
Regular contact with other parents	2.54 ***	.44	.16	1.68	-	3.40
People around here are willing to help neighbors	1.92 ***	.42	.13	1.09	-	2.75
Other caregivers do not understand the struggles I have affording diapers	-1.17 *	.51	-.07	-2.17	-	-.16
Other caregivers judge me as a bad caregiver because I struggle to afford diapers	-1.46 **	.55	-.09	-2.54	-	-.38
I feel alone because I cannot afford enough diapers	-1.99 ***	.58	-.12	-3.12	-	-.86
I feel helpless when I cannot afford enough diapers	-1.00 *	.50	-.07	-1.98	-	-.02
I feel stressed or anxious when I am unable to afford enough diapers	-.05	.48	.00	-1.00	-	.90
The people close to me do not treat me respectfully	-1.49 *	.60	-.08	-2.66	-	-.32
The people close to me are not positive or encouraging to me	-3.06 ***	.57	-.18	-4.18	-	-1.94
Has moved homes at least once in the past year	-1.09 *	.45	-.07	-1.98	-	-.21
Hispanic identity	1.22 *	.54	.06	.15	-	2.28
Black identity	.24	.62	.01	-.97	-	1.45
Asian or another identity	-.61	.62	-.03	-1.83	-	.62
Middle income	.24	.52	.02	-.79	-	1.27
High income	-1.18	.69	-.06	-2.55	-	.18
Divorced, separated, widowed	-1.75	.90	-.06	-3.51	-	.01
Never married	-.93	.61	-.05	-2.14	-	.27
Domestic partnership, civil partnership	-.74	.68	-.03	-2.09	-	.60
No high school degree	.46	1.00	.01	-1.51	-	2.42
Some college	.85	.58	.04	-.29	-	1.98
Two-year degree	.64	.72	.03	-.77	-	2.05
Four-year degree	1.32 *	.66	.07	.02	-	2.62
Post graduate education	.53	.75	.02	-.95	-	2.00
Homemaker employment status	-2.07 ***	.55	-.11	-3.14	-	-.99
Other employment status	-.68	.62	-.03	-1.90	-	.54
Constant	23.93***	​	​	​	​	​
*df*	972	​	​	​	​	​

For the overall model, *R*^2^ = .36, adjusted *R*^2^ = .34. *F*(26, 972) = 20.43, *p* < 0.01.

Notes: Higher ISEL scores indicate higher perceived social support.

Reference groups for demographic characteristics were: white race, low-income, married, high school diploma as highest education achieved, and fulltime or parttime employment status.

*p < 0.05. **p < 0.01. ***p < 0.001.

Receiving diapering guidance and other parenting advice from a nurse or other health professional was something that households with (69.0%, n=322) and without diaper insecurity (67.7%, n=361) alike indicated that they “completely agree” or “agree” they would feel comfortable receiving. There were no group differences in comfort receiving diapering guidance from this source. When asked the extent to which they would feel comfortable accepting diaper assistance from family, friends, diaper banks, or charity, more caregivers with diaper insecurity responded that they “completely agree” or “agree” they would feel comfortable accepting (72.4%, n = 338) compared to households without diaper insecurity (50.3%, n = 268), *X*^*2*^ (1, N = 1,000)=50.896, *p* <.001. Caregivers reported the extent to which receiving 50 free diapers per month, per child, from a local diaper bank would be helpful for their family. Overall, 68.1% (n=639) of study participants indicated this would be “extremely helpful” or “very helpful.” There was not a difference between households with and without diaper insecurity (69.6%, n=316 with diaper insecurity; 66.5%, n=323 without). Lastly, the majority of households, both with diaper insecurity (74.3%, n=336) and without (60.3%, n=285), specified that expanding federal and/or state programs, TANF, or Medicaid to offer financial assistance for the purchase of diapers would be “extremely helpful” or “very helpful.”

## Discussion

Consistent with previous findings, 1 in 2 households with young children in the United States reported diaper insecurity.^6–8^ This proportion (47%) is higher than in the decade prior to the COVID-19 pandemic (36%) and its increase is a concerning indicator of material hardship.^5,11,23^ Material hardship, difficulty meeting material basic needs (e.g. food, housing, healthcare) provides a measure of assessing poverty that is more comprehensive than measuring income alone.^27^ Increased prevalence of diaper insecurity among U.S. households with young children has implications for increased utilization of the healthcare system to treat diaper related illnesses such as diaper dermatitis.^11^ Additionally, diaper insecurity may cause disruptions to the workforce and early child care settings when family members must miss work or childcare if caregivers do not have the diapers required for childcare participation and must stay home to care for a child instead.^3^ The unequal burden on these health, economic, and social risks are problematic. All families deserve to be able to raise their children in safe and supportive conditions.

The consequences of diaper insecurity that may be felt most acutely for individual families are impacts to caregiver mental health.^5,14^ The WHO recognizes mental health “as a state of mental well-bring that enables people to cope with the stresses of life, realize their abilities, learn and work well, and contribute to their community.”^28^ Our findings identified diaper affordability as a stressor for families that was associated with perceptions of social support. Gaps in support have implications for mental well-being and coping with the stressors of parenting young children. The majority of caregivers in our study who endorsed diaper insecurity reported feeling stressed, anxious, and/or helpless when they could not afford diapers for their children. Further, caregivers reported feeling judged as a bad parent related to their struggles affording diapers in addition to having lower perceived social support and feelings that other parents did not understand their diaper affordability challenges. Programs addressing mental health for caregivers of infants and young children should explore opportunities to integrate diaper distribution and partnerships with diaper banks as part of the delivery of maternal mental health services, including services designed to support overall mental well-being without diagnosis of a mental health condition. The coverage of diapers through Medicaid, as is being done in Delaware and Tennessee for example, could present unique policy opportunities to co-locate diaper distribution with the delivery of maternal mental health services in community-based and clinical contexts.^29,30^ Supporting the overall mental well-being of caregivers, instead of intervening only during a period of crisis or in response to a specific diagnosis, is important for addressing the documented stress tied to parenting.^1^

The vast majority of households in our study with diaper insecurity reported accessing one or more resources for help meeting their basic needs in the last year. Currently, U.S. policies are structured so that none of the safety net resources available to families with young children support all of a household’s unmet basic needs. Rather, resources exist to supplement specific unmet needs such as healthcare or food. This design means that accessing resources can be time-consuming when families must visit multiple places to address multiple basic needs. Basic needs banks, including diaper banks, aim to provide resources alongside other supports to reduce the amount of time households spend meeting their basic needs.^18^

In our study, the prospect of receiving 50 free diapers per month per child from a local diaper bank was helpful for households, both those with and without diaper insecurity. Diapers can be a tool for connecting families to other resources, trust building with healthcare professionals and service providers, and supporting caregivers of young children in a way they have indicated would be meaningful for them.^18^ The fact that the majority of households who did not endorse diaper insecurity based on the three screening questions in our study still reported that receiving free diapers would be helpful suggests that not only are diapers a universal need for young children, but many more families in the U.S. may be struggling to meet material basic needs than society realizes. Among households who did not endorse diaper insecurity, half were comfortable accepting diaper bank support and more felt it would be helpful. This highlights that there are many families in need who would find the support helpful who do not feel comfortable accepting assistance. Therefore, offering resources universally and without judgment is important for supporting holistic well-being and health. The potential to ‘lead with resources’ instead of the conventional approach of first screening for adverse health-related social needs would bypass the gaps identified in this study.^31^ Universal patient education about resources would eliminate patient concerns of potential negative consequences of disclosure reported in other research.^31^

Resources offering assistance with food were the most commonly accessed resources among the households in our sample. It is well documented that families who experience food insecurity are likely to experience diaper insecurity.^12,14,32^ As such, many diaper banks partner with food banks and WIC clinics to provide diaper assistance alongside food assistance.^18^ Including diaper support in state and federal policy programs provides an opportunity to meet families where they are already seeking and receiving assistance. The majority of households in our sample, both those with and without diaper insecurity, indicated that the expansion of these programs to offer diaper assistance would be helpful. It is notable that diaper insecurity is only one component of co-occurring material hardships families may face.^4,14^ Although addressing diaper insecurity is positively associated with addressing other forms of material hardship, it is important to note that addressing diaper insecurity will not eliminate all forms of material hardship for a family.^9,12,15^

Hospitals and health clinics were sometimes accessed by families seeking help meeting their basic needs. The majority of study participants reported comfort receiving diapering guidance and other parenting advice from healthcare professionals. Given the frequency of healthcare visits during infancy and early childhood and the importance of postpartum and primary care, healthcare professionals and healthcare settings offer an opportunity to screen for diaper insecurity then offer diaper aid in partnership with local diaper banks.^9,33,34^ A free diaper insecurity screening module exists in the Epic electronic health records system, but must be activated by the healthcare system to implement. Alongside initiatives to expand screening, institutional and health care team readiness to address patient priorities is critical.

Participants with diaper insecurity were less likely than participants without to feel encouraged or treated in the ways they deserved by healthcare professionals, community organizations, and the people close to them. In any support setting, word of mouth matters—people share with others about the availability of resources and how they were treated. People will not return or encourage others to go if they do not feel respected by staff, institutional policies, or procedures. Ensuring that all families in the U.S. with young children feel respected and supported requires ensuring that all caregivers have access to the material basic needs required to participate in community, the workforce, and in early childhood education settings.

A lack of social support can be isolating, so too can diaper insecurity. Among caregivers in our study, negative affective experiences related to diaper affordability were associated with lower perceptions of social support. Diaper support may bolster perceived social support by providing diapers which can then facilitate community and workforce participation and the social and community interactions that come with such engagements. In our study, regular contact with other parents of young children was a significant predictor of higher perceived social support scores. Having enough diapers to allow for diaper changes while away from home is important for the ability to socialize with other families and thus experience the benefits of social support. In the United States and across the world, many caretaking responsibilities fall to women, through both physical acts of child rearing and the invisible mental load of managing a household with young children.^35^ Diaper support for households with young children is one component of addressing women’s overall health and mental well-being.

### Limitations

The present study uniquely documents the intersection of diaper insecurity and affective components of material hardship. Data were collected via a cross-sectional survey. From the survey we learned how people felt in that moment, their experiences with resources, supports, and healthcare in the last 12 months, and what they thought would be helpful. We did not calculate a power analysis for sample size before the start of the study. Although participants are a nationally representative sample, those without reliable internet access and people who prefer a language other than English could not participate. In our sample, 44% of households with young children reported incomes <200% of the federal poverty guideline. Another source using U.S. census data reported the percentage of U.S. households with children under age 18 with income <200% of the federal poverty guideline to be 37% in the same year our sample was collected.^36^ To date, no screening question has been validated for diaper insecurity. Additionally, future studies are needed to test the impact of diaper distribution interventions on caregiver loneliness, social connectedness, and mental health to determine the extent to which they are helpful and why.

## Conclusion

Many more families in the United States may be struggling to afford diapers than are identified with diaper insecurity screening questions. Caregivers identified negative affective components of diaper affordability including feelings of judgement and aloneness which were associated with lower perceptions of social support. Universally, households with and without diaper insecurity indicated that receiving 50 free diapers per month per child from a local diaper bank would be helpful. The support and expansion of diaper banks’ distribution efforts to serve more households would help families in ways they have identified as acceptable and meaningful.

## Supplemental material

Supplemental material - Caregiver report of the experiences of infant and child diaper insecurity, social support, and utilization of resources to help meet basic family need in the United States: A nationally-representative cross-sectional survey Supplemental material for Caregiver report of the experiences of infant and child diaper insecurity, social support, and utilization of resources to help meet basic family need in the United States: A nationally-representative cross-sectional survey by Kelley E. C. Massengale, Megan V. Smith and Kristin P. Tully in Women's Health.

## Data Availability

The raw data are available from the corresponding author upon reasonable request.*
